# 17β-Estradiol Promotes Apoptosis of HepG2 Cells Caused by Oxidative Stress by Increasing Foxo3a Phosphorylation

**DOI:** 10.3389/fphar.2021.607379

**Published:** 2021-03-15

**Authors:** Yusheng Guo, Xiangsheng Cai, Hanwei Lu, Qiqi Li, Ying Zheng, Zefang Lin, Zexiong Cheng, Maoxiang Yang, Li Zhang, Lei Xiang, Xiaorong Yang

**Affiliations:** ^1^ Clinical Laboratory, First Affiliated Hospital/School of Clinical Medicine, Guangdong Pharmaceutical University, Guangzhou, China; ^2^ Department of Medical Laboratory, School of Clinical Medicine, Guangdong Pharmaceutical University, Guangzhou, China; ^3^ Center for Medical Experiments, University of Chinese Academy of Science-Shenzhen Hospital, Shenzhen, China; ^4^ Department of Integrative Chinese and Western Medicine, The First Affiliated Hospital of Guangdong Pharmaceutical University, Guangzhou, China

**Keywords:** 17β-estradiol, hepatocellular carcinoma, oxidative stress, apoptosis, foxo3a phosphorylation

## Abstract

Liver cancer is associated with high mortality, particularly in patients infected with the hepatitis B virus. Treatment methods remain very limited. Here, we explored the effects of 17β-estradiol (E2) on apoptosis of various liver cell lines (LO2, HepG2, and HepG2.2.15 cells). Within a certain concentration range, 17β-estradiol induced oxidative stress and apoptosis of HepG2 cells, downregulated ERα-36 expression, and increased Akt and Foxo3a phosphorylation. *p*-Foxo3a became localized around the nucleus but did not enter the organelle. The levels of mRNAs encoding manganese superoxide dismutase (MnSOD) and catalase, to the promoters of which Foxo3a binds to trigger gene expression, were significantly reduced in HepG2 cells. 17β-estradiol had no obvious effects on LO2 or HepG2.2.15 cells. We speculate that 17β-estradiol may induce oxidative stress in HepG2 cells by increasing Foxo3a phosphorylation, thus promoting apoptosis. This may serve as a new treatment for hepatocellular carcinoma.

## Introduction

Liver cancer is common and associated with high mortality (Mak et al., 2018); hepatocellular carcinoma (HCC) is the most common cancer type (Liu and Suo, 2020). Chronic HBV infection is an important risk factor for HCC (Nayagam et al., 2020). Traditional treatments include surgical resection, liver transplantation, local ablation, and transcatheter arterial chemoembolization. The clinical effects on, and prognoses of, patients with advanced liver cancer are very poor; more effective therapies are needed (Bruix et al., 2019). Primary liver cancer occurrence and development is complex, involving multiple factors and several regulatory steps. Molecularly targeted therapy exploiting the abnormal signaling pathways of primary liver cancer are being intensively researched (Rossetto and De Re, 2019), However, as cancer occurrence and development remain poorly understood, and as the pathophysiological mechanism remains unclear, such therapies are not yet advanced. Sorafenib was the first such oral drug approved by the European Medicines Agency and Food and Drug Administration to treat primary liver cancer. However, sorafenib used as first-line treatment for HCC exhibits several side-effects, high-level drug-resistance development, and is associated with poor patient tolerance (Moawad et al., 2020).

Epidemiologically, the incidence of, and mortality from, liver cancer in males are much higher than in females (Mak et al., 2018), suggesting that estrogen may be protective. Estrogen regulates reproductive physiology and function (Satirapod et al., 2020), cell growth and proliferation, apoptosis, the immune response, and metabolism (Guerra et al., 2020; Jehle et al., 2020). The liver is targeted by estrogen (Clegg et al., 2017; Kur and Kolasa-Wołosiuk, 2020). Estrogen and estrogen receptor (ER) agonists inhibit the growth of liver cancer HepG2 cells by downregulating proliferation and promoting apoptosis (Shen et al., 2018). Estrogen binds to its receptor to inhibit the activity of NF-KB, thereby regulating tumor growth (Pelekanou et al., 2016). Thus, the estrogen signal transduction pathway (involving the Erα receptor and estrogen *per se*) may inhibit the occurrence and development of liver cancer.

Chronic HBV infection is an important risk factor for HCC in our country; we encounter many such patients. Here, we used HepG2.2.15 cells harboring the hepatitis B virus to explore whether estrogen might exert a therapeutic effect. LO2 cells are human normal liver cells derived from human embryos; HepG2 cells were derived from the liver cancer tissue of a 15-year-old Caucasian teenager and are widely used in liver cancer-related *in vitro* studies; the HepG2.2.15 cell line is derived from the HepG2 human liver cancer cell line transfected with HBV genes (Xia et al., 2016; Li et al., 2020). Therefore, we explored the effects of 17β-estradiol on LO2, HepG2, and HepG2.2.15 cells, and the mechanisms in play, to derive a theoretical basis for biotherapy of hepatomas with 17β-estradiol.

## Materials and Methods

### Reagents

17β-estradiol, dihydroethidium (DHE), and 2′,7′-dichloro dihydro fluorescein diacetate (DCFH-DA) were purchased from Sigma-Aldrich (St. Louis, MO, United States). Antibodies (rabbit anti-human) against ERα, Akt, *p*-Akt, Foxo3a, *p*-Foxo3a, and β-actin were purchased from Cell Signaling Technology (Beverly, MA, United States). The 17β-estradiol was dissolved in dimethyl sulfoxide (DMSO) to prepare a stock solution at a concentration of 50 mg/ml, and was stored at −20°C. Complete Dulbecco’s modified Eagle medium (DMEM; Gibco, Gaithersburg, MD, United States) was added to dilute the 17β-estradiol to the appropriate concentrations prior to use.

### Cell Culture and Treatment

LO2, HepG2, and HepG2.2.15 cells were cultured in DMEM supplemented with 10% fetal bovine serum (Gibco or CellMax, Sunnyvale, CA, United States) at 37°C in a humidified atmosphere of 5% CO_2_. After digestion and counting, cells were seeded into 96-well plates (5,000 cells/well) or 6-well plates (50,000 cells/well). When grown to 70–80% confluence, 17β-estradiol was added to various levels and the cells were cultured further until they were used for the MTT assay or other detection methods.

### MTT Assay

First, 17β-estradiol of different concentrations was added to each well, and the cells were cultured for 24, 48, or 72 h, followed by incubation with 5 mg/L MTT for 0.5–4 h. Then, the medium was discarded and 200 μL of DMSO was added to each cell. Finally, absorbance at a wavelength of 490 nm was measured using a microplate reader (Multiscan-GO, Thermo Fisher Scientific, Waltham, MA, United States).

### qPCR and Ribonucleic Acid-Seq Analysis

Total RNA was isolated using the TRIzol reagent and reverse transcribed into cDNA using a commercial kit (Takara (Dalian), Shiga, Japan). Relative mRNA abundances were calculated as the differences between the thresholds of target genes and the housekeeping gene (GAPDH). The average of three replicates was calculated. The primers are listed in Table 1.

**TABLE 1:** The primers used in the qPCR.

Name	Primers sequence (5′-3′)
MnSOD-forward	ACA​AAG​ATG​GTG​TGG​CCG​AT
MnSOD-reverse	AAC​GAC​TTC​CAG​CGT​TTC​CT
Catalase-forward	AGT​GAT​CGG​GGG​ATT​CCA​GA
Catalase-reverse	GAG​GGG​TAC​TTT​CCT​GTG​GC
GAPDH-forward	GAA​GGT​GAA​GGT​CGG​AGT​C
GAPDH- reverse	GAA​GAT​GGT​GAT​GGG​ATT​TC

Construction of the cDNA library, Illumina sequencing (paired-end on Illumina HiSeqTM2000), and RNA-Seq analysis were performed by a commercial gene-sequencing company (Gene Denovo, Guangdong, China). Low-quality sequence reads with >5% N bases and reads containing adaptor sequences were removed before data analysis. The sequencing data are available on the NCBI BioProject website (accession no. PRJNA664533). Sequencing reads were mapped to the reference sequence using SOAPaligner/soap2 (Li et al., 2009). The gene expression level was measured by the number of uniquely mapped reads per kilobase of exon region per million mappable reads (RPKM; RPKM = 106C/(NL/103), where C is the number of reads uniquely mapped to the given gene, N is the number of reads uniquely mapped to all genes, and L is the total length of exons from the given gene). For genes with more than one alternative transcript, the longest transcript was selected to calculate the RPKM. All statistical analyses and visualization with expression data were conducted using R software (http://www.r-project.org/). After the expression level of each gene was calculated, differential expression analysis was conducted using edgeR (Robinson et al., 2010). The false discovery rate (FDR) was used to determine the *p*-value threshold for multiple tests, and for the analysis, FDR ≤0.001 and an absolute log2 ratio value ≥1 were used to determine the significance of differences in gene expression. Gene Ontology (GO) enrichment analysis provides all GO terms that significantly enriched in the differentially expressed genes (DEGs) comparing to the genome background and filters the DEGs that correspond to biological functions. GO enrichment analysis was conducted using OmicShare tools.

### Reactive Oxygen Species Detection

After treatment with 17β-estradiol, cells were washed with PBS and stained with DHE or DCFH-DA for 10–90 min in the dark at 37°C or room temperature. Then the cells were observed under a fluorescence microscope (Olympus FV1000, Japan) and analyzed using Image-Pro Plus software after being washed with PBS. Fluorescence intensity was determined using flow cytometry (BD Biosciences, San Jose, CA, United States) after cells were trypsinized and collected.

### Detection of Apoptosis by Flow Cytometry

Apoptosis was detected with the aid of an AnnexinV-FITC/PI Kit (KeyGen, Nanjing, China). After trypsin digestion and resuspension in PBS, 1 × l05 cells/group were mixed with Annexin V-FITC (5 μL) and propidium iodide (5 μL) in turn, and incubated in the dark for 15 min. Flow cytometry (BD Biosciences, San Jose, CA, United States) followed.

### Immunofluorescence Staining

Cells were fixed in 4% (w/v) paraformaldehyde for 20 min and washed with PBS three times for 5 min each time. After treatment with 0.2% (v/v) Triton X-100 for 15 min, the cells were incubated with goat serum at 37°C for 30 min and the medium discarded. The first antibody (1:100 dilution) was added, and the cells washed with PBS three times at 4°C for 5 min each time. Next, 1:100-diluted Cy3-labeled donkey anti-mouse secondary antibody and FITC-labeled goat anti-rabbit secondary antibody were added, followed by incubation for 60 min at 37°C. After washing the slides with PBS, DAPI staining solution was added; the nuclei were stained at 37°C for 15 min and then washed twice with distilled water for 5 min each time. The fluorescence buffer did not contain glycerin. Six random fields were imaged using a fluorescence microscope and the fluorescence intensity analyzed with the aid of Image-Pro plus ver. 6.0 software.

### Western Blotting

Samples were lyzed in RIPA buffer, homogenized, cracked, and centrifuged to obtain total protein extracts; protein concentrations were measured with the bicinchoninic acid assay. Proteins were subjected to SDS-PAGE and then transferred to PVDF membranes under a constant current (200 mA for 1 h). The membranes were placed into TBST sealing solution with 5% (w/v) skim milk powder for 1 h, the primary antibody added, followed by shaking at 4°C overnight. After washing, the second antibody was added and incubation proceeded at 37°C for 1 h. After washing, the light-emitting solution was added; and the membranes compressed, exposed, and fixed. A Labworks model 4.5 image acquisition and analysis system was used to determine the optical densities of protein bands. Actin served as the internal standard. Each experiment was repeated at least three times.

### Statistical Analysis

SPSS ver. 20.0 software was used to analyze data and GraphPad Prism ver. 6.0 software for statistical mapping. The ANOVA test was employed to analyze data and the chi-squared test for comparisons. A *p*-value <0.05 was taken to indicate statistical significance.

## Results

### Effects of 17β-Estradiol on Human Hepatocyte Proliferation

**FIGURE 1:**
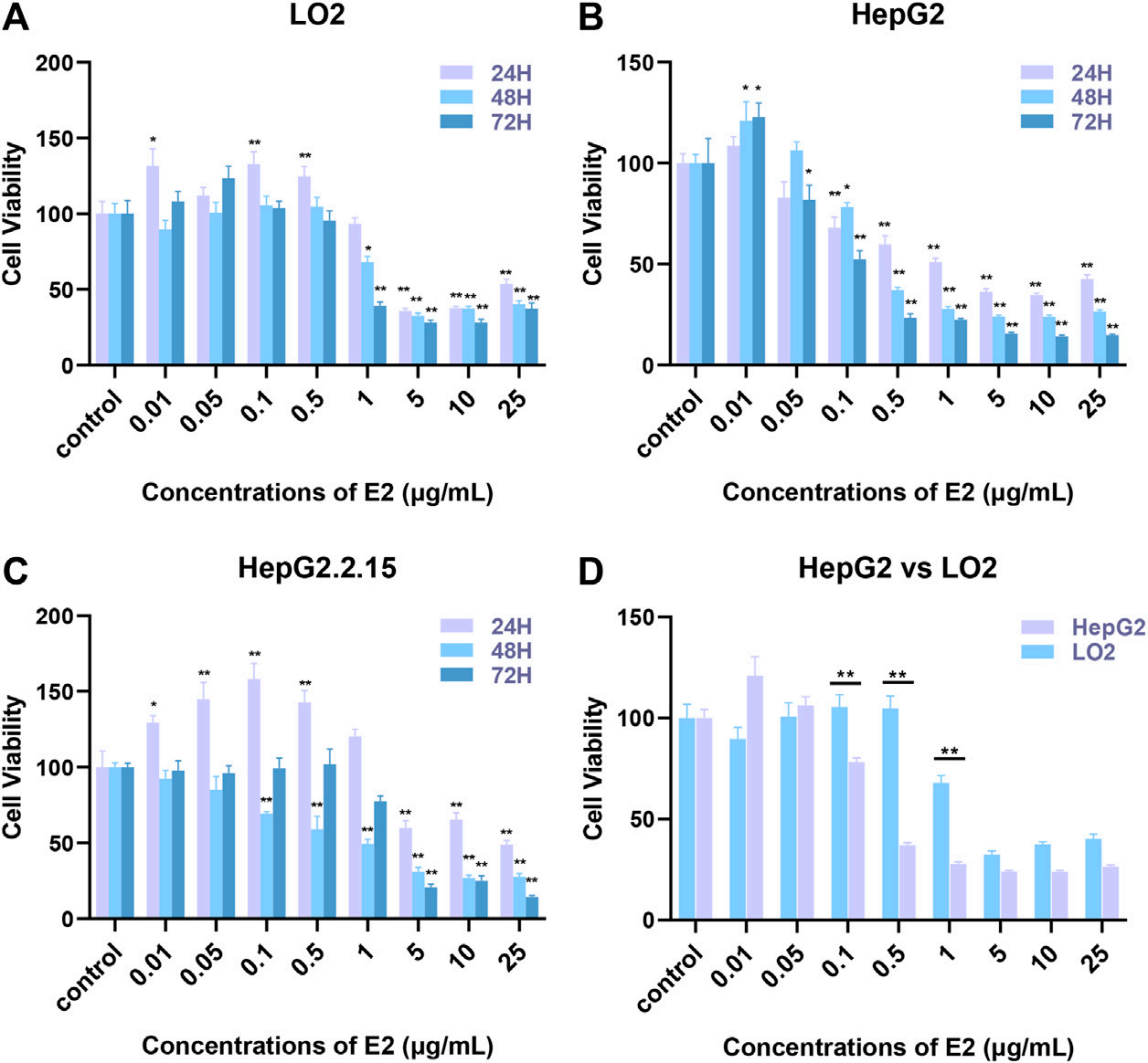
The effects of 17β-estradiol on the proliferation of HepG2, HepG2.2.15, and LO2 cells. **(A–C)** The histograms show the effects of various levels of the hormone at different times, compared to control groups. *p < 0.05, **p < 0.01. **(D)** The histogram compares HepG2 and LO2 cell proliferation during 17β-estradiol treatment over 48 h, **p < 0.01.

HepG2, HepG2.2.15, and LO2 cells were treated with 17β-estradiol (0.01 μg/ml to 25 μg/ml) for 24, 48, and 72 h (Figure 1A). 17β-estradiol at 0.01–0.5 μg/ml had no effect on LO2 cell proliferation but significantly inhibited proliferation at 5–25 μg/ml (*p* < 0.01). Figure 1B shows that 17β-estradiol had no effect on HepG2 proliferation at 0.01 μg/ml but significantly inhibited proliferation at 0.1–25 μg/ml at 24, 48, and 72 h (Figure 1B, *p* < 0.01). The IC_50_ values were 0.7968, 0.3446, and 0.1256 μg/ml, respectively, at these times. For HepG2.2.15 cells, 17β-estradiol at 0.01–1 μg/ml did not inhibit proliferation to 24 or 72 h, but significantly inhibited proliferation at 1–25 μg/ml. However, at 48 h, 17β-estradiol at levels over 0.1 μg/ml significantly inhibited proliferation (Figure 1C, *p* < 0.01). To explore whether the inhibitory effect of 17β-estradiol was tumor-specific, we compared the inhibitory effects of 17β-estradiol on HepG2 and LO2 cells at 48 h. The results (Figure 1D) showed that the inhibitory effect of 17β-estradiol on HepG2 cells was significantly higher than that on LO2 cells at relatively low concentrations (0.01–1 μg/ml). 17β-estradiol thus strongly inhibited hepatoma cell proliferation in a tumor-specific manner.

### 17β-Estradiol Promotes Apoptosis of HepG2 Cells

**FIGURE 2:**
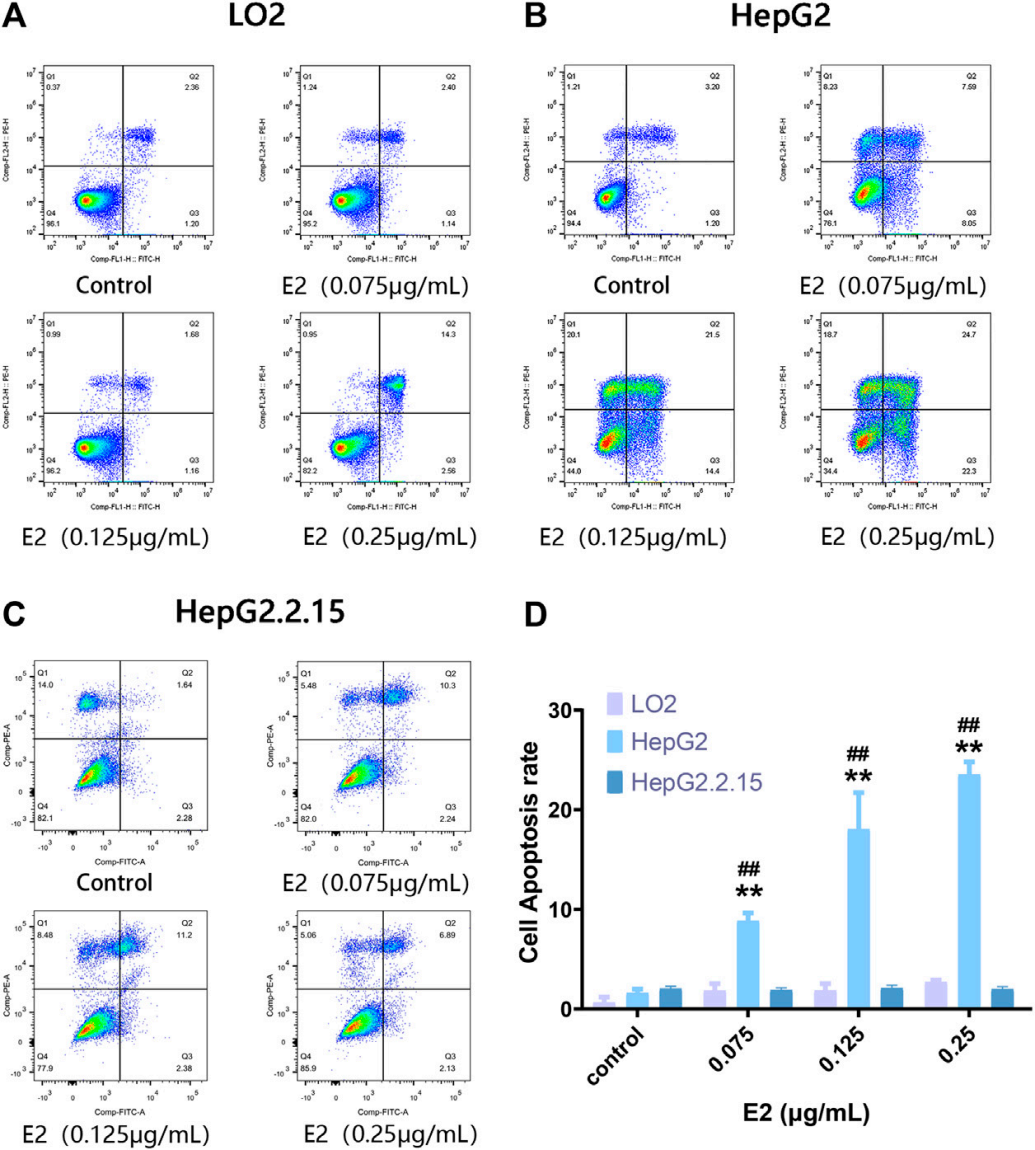
The extent of apoptosis of HepG2, HepG2.2.15, and LO2 cells treated with 17β-estradiol. **(A–C)** Tetrad plots showing the effects on the three cell types after 48 h. Annexin V-FITC/PI double-staining was used to measure the proportions of apoptotic cells. **(D)** The histogram shows the apoptotic proportions of HepG2 and LO2 cells. All experiments were performed in triplicate. **p < 0.01 compared to the control group, ^##^ *p* < 0.01 compared to LO2 cells at the same dose.

To explore a possible specific apoptotic effect of 17β-estradiol on HepG2 cells, we used the Annexin V-FITC/PI double-staining method to evaluate apoptosis (via flow cytometry) after treatment of cells with 7β-estradiol for 48 h. Compared to the control groups, 17β-estradiol did not induce apoptosis of LO2 or HepG2.2.15 cells (Figures 2A,C). 17β-estradiol dose-dependently induced significant apoptosis of HepG2 cells by 48 h (Figure 2B); the apoptotic cell percentages are compared in Figure 2D. The results of Annexin V-FITC/PI double-staining were consistent with these results; 17β-estradiol specifically inhibited hepatoma cell proliferation and triggered apoptosis.

### 17β-Estradiol Increases Oxidative Stress in HepG2 Cells

**FIGURE 3:**
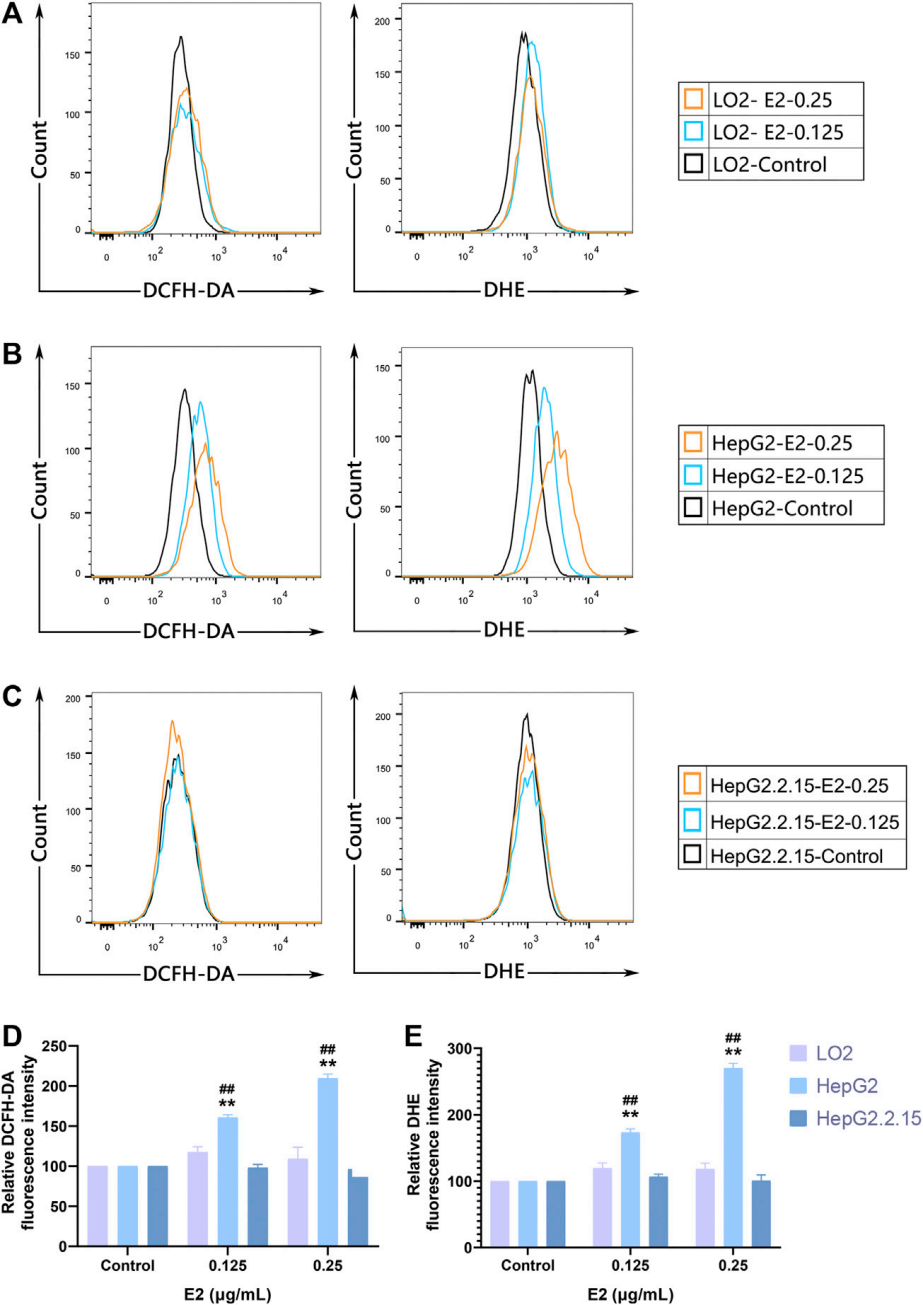
ROS accumulation in cells as assessed by flow cytometry. **(A–C)** Fluorescent ROS peaks of HepG2, HepG2.2.15, and LO2 cells, respectively, induced by 17β-estradiol. After treatment with 17β-estradiol for 48 h, cells were stained using DCFH-DA and DHE fluorescent probes and examined by flow cytometry. **(D,E)** The histograms show the differences in the ROS levels of HepG2 and LO2 cells. All experiments were performed in triplicate, **p < 0.01 compared to the control group, ##*p* < 0.01 compared to LO2 cells at the same dose.

**FIGURE 4:**
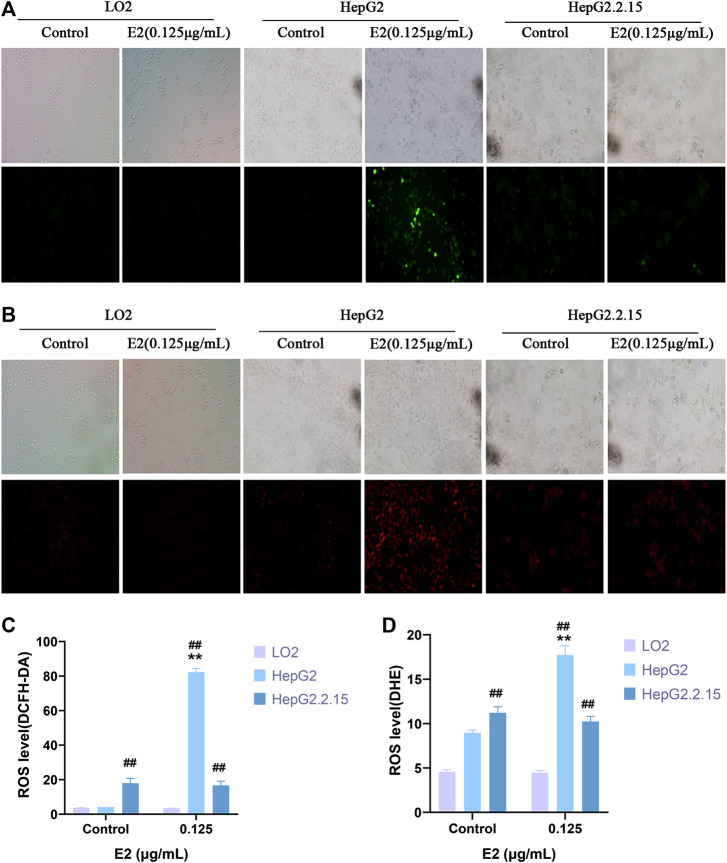
ROS levels in cells as evaluated by immunofluorescence staining. **(A,C)** DCFH-DA. **(B,D)** DHE. Fluorescence microscopy was used to observe and record fluorescence levels. **p < 0.01 compared to the control group, ^##^ *p* < 0.01 compared to LO2 cells at the same dose.

We used the DCFH-DA and DHE fluorescent probes to explore whether 17β-estradiol induced oxidative stress in hepatoma cells. After treatment with 17β-estradiol for 48 h, the probes were used for *in vitro* staining; fluorescence intensity detected by flow cytometry reflected the levels of intracellular ROS (Figures 3A–C). As shown in Figures 3D,E, compared to control LO2 cells, the ROS level in HepG2 cells was significantly increased by treatment with 17β-estradiol for 48 h (*p* < 0.01). Under the fluorescence microscope, HepG2 cells (compared to controls) exhibited obvious green or red fluorescence (Figure 4). This further confirmed that 17β-estradiol increased ROS levels in HepG2 cells.

### Changes in ERα/Akt/Foxo3a Signaling Induced by 17β-Estradiol

**FIGURE 5:**
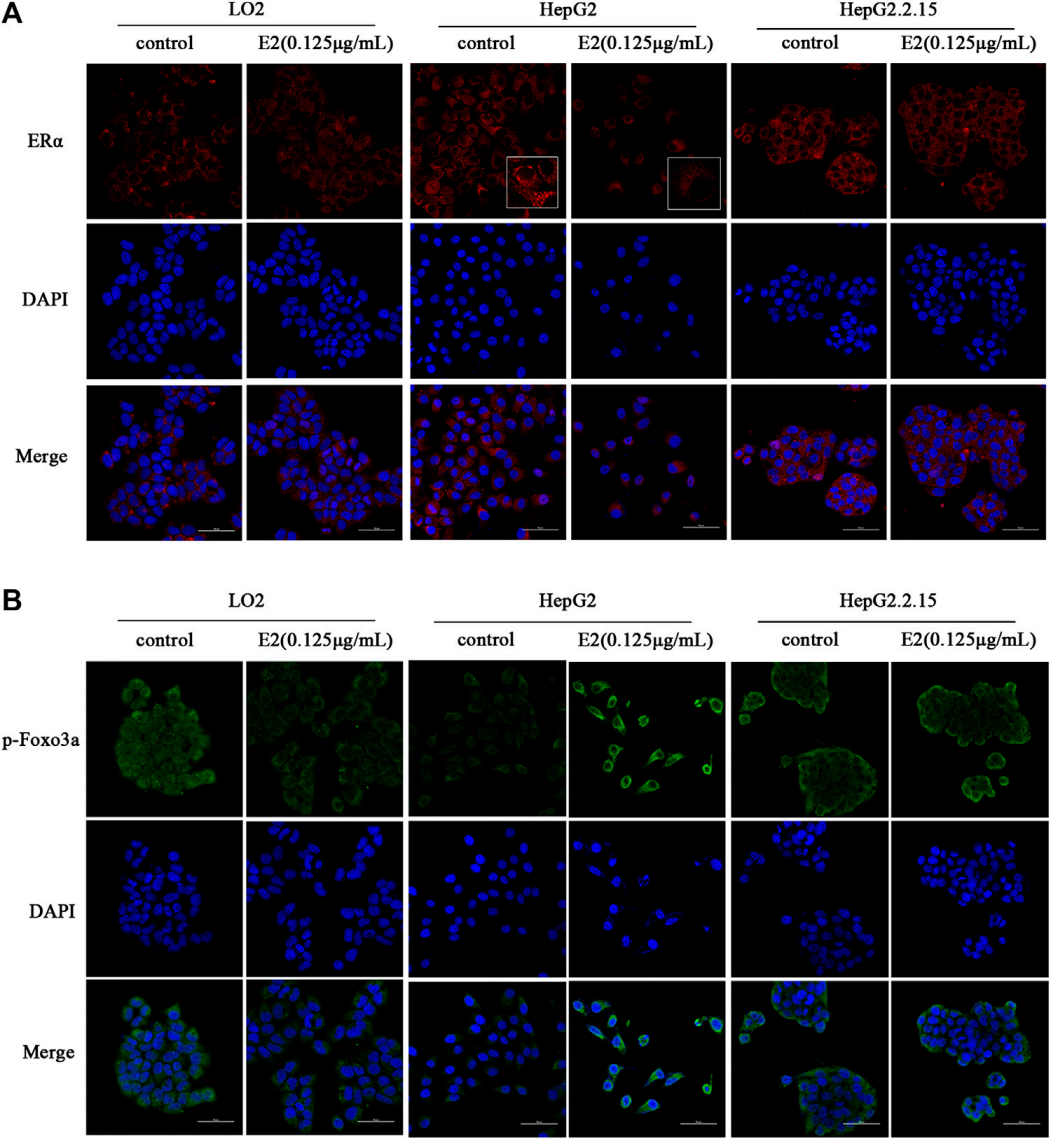
The effects of 17β-estradiol (0.125 μg/ml for 48 h) on ERα **(A)** and *p*-Foxo3a **(B)** expression and localization. The effects on HepG2, HepG2.2.15, and LO2 cells, respectively, as revealed by fluorescence microscopy.

ERα and *p*-Foxo3a levels were evaluated by immunofluorescence staining. As shown in Figures 5A,B, after treatment with 17β-estradiol for 48 h, ERα was downregulated in HepG2 cells, but the level of phosphorylated Foxo3a increased and the protein was localized around the nucleus (Figure 5B). The phosphorylated protein could not enter the nucleus to trigger the expression of Mn-SOD and catalase. ERα and phosphorylated Foxo3a levels in LO2 and HepG2.2.15 cells were not affected by 17β-estradiol treatment, and the Foxo3a protein was not significantly localized around the nucleus.

**FIGURE 6:**
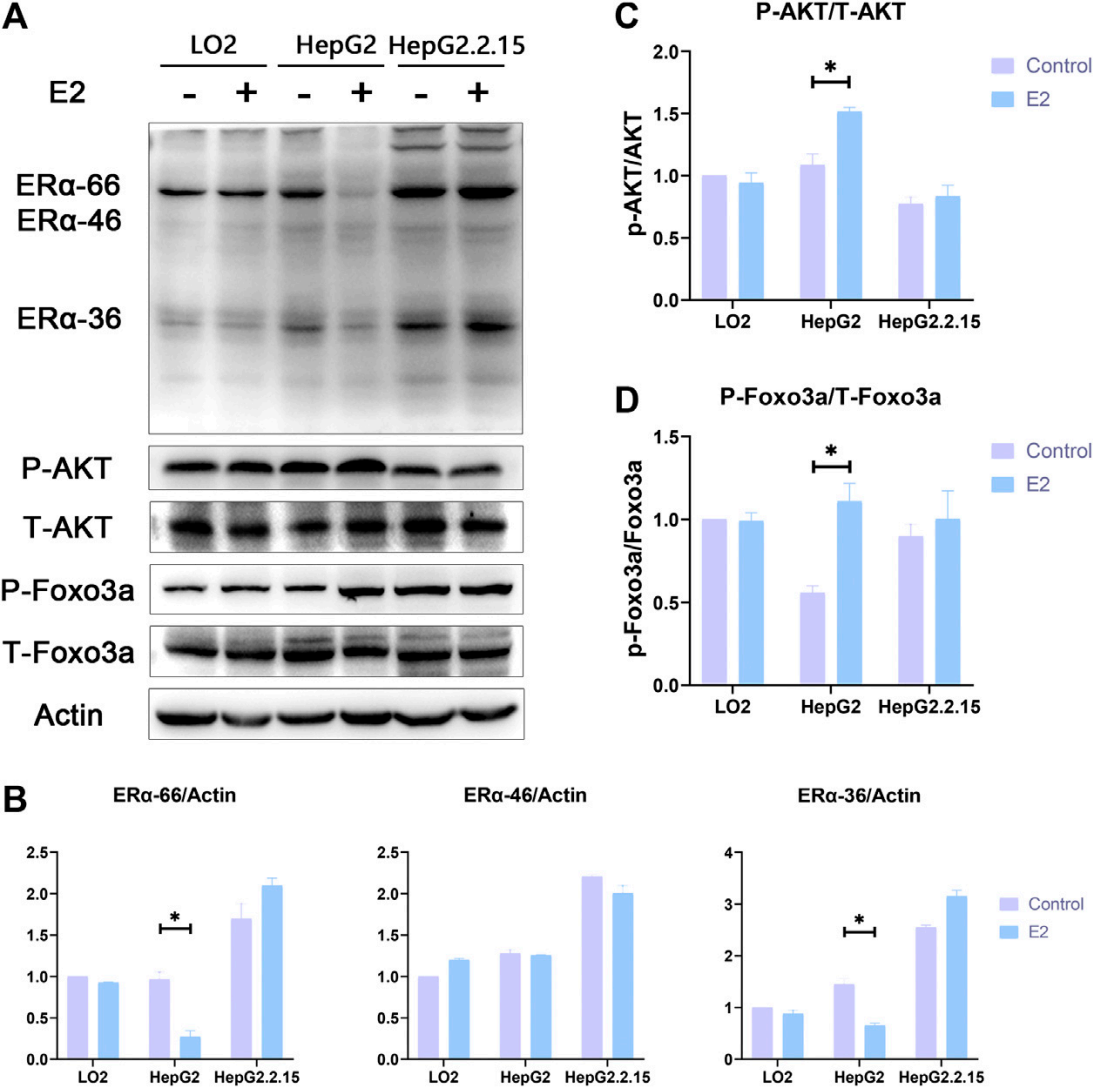
**(A)**. The effects of 17β-estradiol on the expression levels of ERα, *p*-Akt, *p*-Foxo3a, ERα-36, t-Akt, and t-Foxo3a in HepG2, HepG2.2.15, and LO2 cells treated with 17β-estradiol (0.125 μg/ml) for 48 h, as revealed by Western blotting. **(B-D)** The effects of 17β-estradiol on ERα-36, ERα-46, ERα-66, *p*-Akt/t-Akt, and *p*-Foxo3a/t-Foxo3a levels, respectively. *p < 0.05 compared to the control groups.

Western blotting showed that the levels of ERα-66 and ERα-36 in HepG2 cells were significantly reduced after 24 h of 17β-estradiol treatment (both *p* < 0.05); such treatment notably increased the levels of phosphorylated AKT and Foxo3a (both *p* < 0.05). The levels of ERα-66 and ERα-36 in LO2 and HepG2.2.15 cells were not affected after 24 h of 17β-estradiol treatment. ERα-46 levels in the three liver cell lines were not affected after 24 h of 17β-estradiol treatment. The *p*-Akt level did not change in the LO2 cells and HepG2.2.15 cells, and *p*-Foxo3a expression was not affected in LO2 and HepG2.2.15 cells after 24 h of 17β-estradiol treatment (Figure 6).

### Changes in the Levels of Enzymes Related to Oxidative Stress Induced by 17β-Estradiol

Transcriptome sequencing was performed to an average depth of 48 million reads per sample using 150-base, paired-end reads. Quality control analysis indicated that the Phred nucleotide quality score was >30 (indicating 95% accuracy of the base call) across the length of the reads. The number of trimmed reads ranged from 3.8 to 6.1 × 10^7^ with an average of 84% of reads mapping to the *Homo sapiens* genome. A total of 17,860 known mRNAs in cells were detected quantitatively. And 3,682 differentially expressed mRNAs were obtained.

**FIGURE 7:**
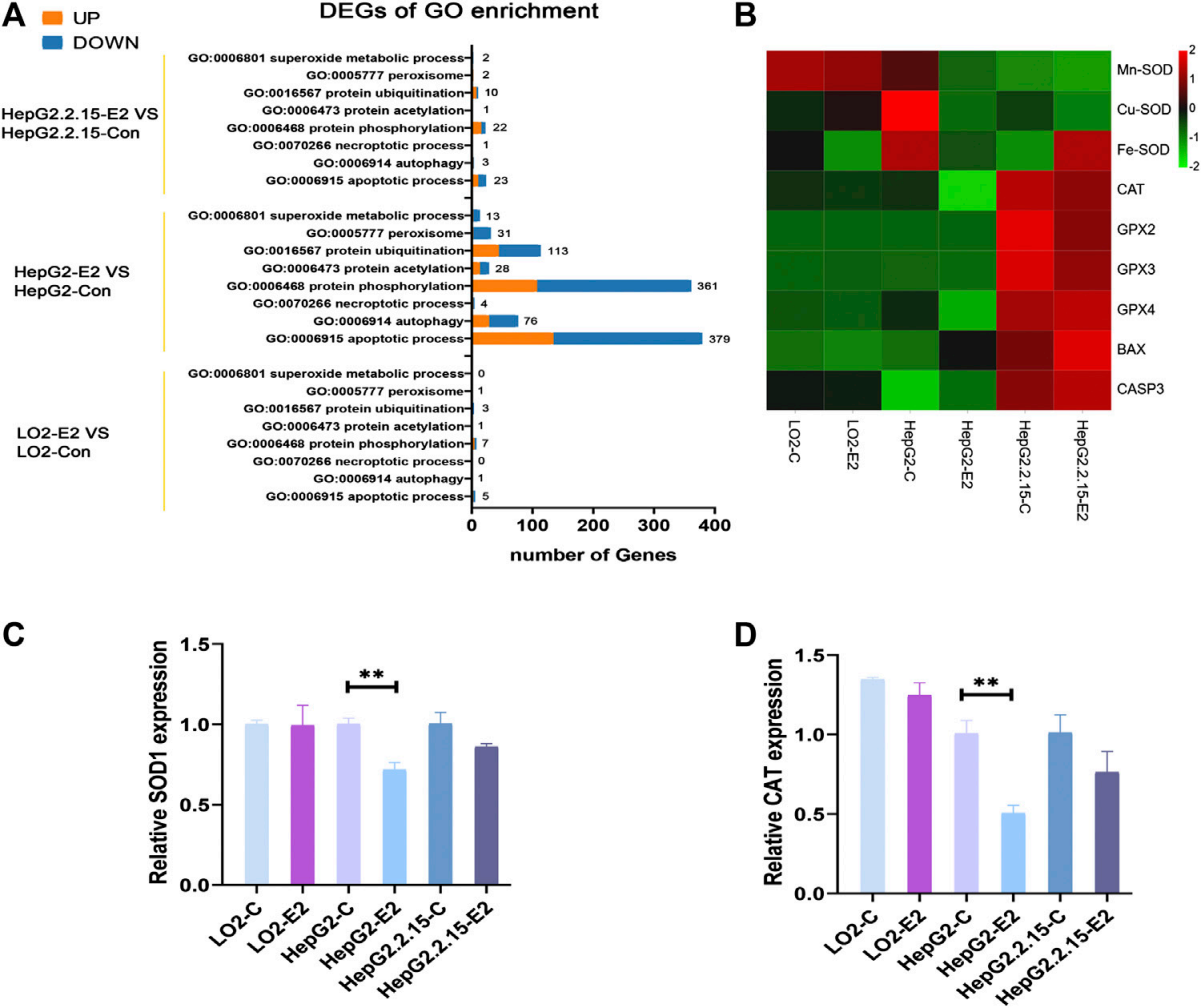
17β-estradiol decreased the expression levels of SODs and catalase in HepG2 cells. **(A)** Enriched Gene Ontology (GO) biological process **(B)** A heatmap of the enzyme expression levels in the three cell lines. qRT-PCR was used to measure the expression levels of mRNAs encoding SOD1 **(C)** and catalase **(D)** in HepG2 cells. **p < 0.01 compared to the control group.

Then, we performed an analysis on the GO terms of DEGs in three cell lines with E2 treatment. The DEGs in HepG2-E2 groups were mainly concentrated in the following biological processes: “apoptotic process”, “protein phosphorylation”, “peroxisome”, “superoxide metabolic process”, “autophagy”, and “protein ubiquitination”. Besides, DEGs in the“peroxisome” and “superoxide metabolic process” were significantly downregulated. There is no particular functional class of genes overrepresented in DEGs in LO2-E2 group and HepG2.2.15-E2 group (Figure 7A).

To verify that 17β-estradiol increased Foxo3a phosphorylation in HepG2 cells, rendering the protein unable to enter the nucleus to initiate transcription of Mn-SOD and catalase, we analyzed the expression levels of enzymes associated with oxidative stress. The heatmap showed that Mn-SOD, Cu-SOD, Fe-SOD, and catalase were downregulated in HepG2 cells after 17β-estradiol treatment (Figure 7B). To confirm these results, we employed RT-qPCR to analyze the mRNA expression levels of Mn-SOD and catalase. As shown in Figures 7C,D, after addition of 17β-estradiol, the Mn-SOD (SOD1) level in HepG2 cells became significantly higher than that in control cells. However, the levels of mRNAs encoding Mn-SOD and catalase in LO2 cells did not change and, indeed, decreased slightly in HepG2.2.15 cells.

## Discussion

Liver cancer is common and is associated with a high mortality rate. Several molecular-targeting drugs have been used as treatments (Dabney et al., 2019; Anwanwan et al., 2020); sorafenib was the first such oral drug to be approved. However, sorafenib exhibits several side-effects, and is associated with high-level drug-resistance and poor patient tolerance. It is essential to discover targeted drugs that are more efficacious, have fewer side-effects, and are cheaper (Dutta and Mahato, 2017). The aims of this study were to observe differences in the apoptosis-promoting effects of 17β-estradiol in three liver cell lines and to determine the mechanism by which 17β-estradiol promotes HepG2 apoptosis, to aid the development of hepatoma drugs targeting this pathway. 17β-estradiol expression affects breast cancer development (Sreekumar et al., 2020); the effects of 17β-estradiol on liver cancer and other tumors is less well understood. We found that 17β-estradiol (at different concentrations) promoted apoptosis of HepG2 cells, but not LO2 or HepG2.2.15 cells. To the best of our knowledge, this is the first such report. Because the concentration of the 17β-estradiol drug was high, we will consider administering it via local interventions according to current interventional treatments for liver cancer in future studies.

We evaluated the mechanism underlying the effect of 17β-estradiol on apoptosis in HepG2 cells and found that 17β-estradiol increases the ROS levels in HepG2 cells through flow cytometry analysis and immunofluorescence staining. Oxidative stress can cause apoptosis (Wang et al., 2015). 17β-estradiol had no obvious effects on LO2 or HepG2.2.15 cells. To the best of our knowledge, this is the first report on oxidative stress induced by 17β-estradiol in three liver cell lines.

We then explored how 17β-estradiol induced oxidative stress. There are two estrogen signal transduction pathways, the classical pathway mediated by the estrogen nuclear receptor (*ner*, including ERα-66 and ERβ) and the rapid pathway mediated by the estrogen membrane receptor (GRER, EGFR, ERα-36). The former is a slow-response pathway that functions via genomic effects; the latter is a fast-reaction pathway that operates by changing protein conformations, and is also termed the stress conduction pathway (Zhao et al., 2019). ERα-36 rapidly activates non-genomic signaling pathways when it binds to estrogen-related receptors (ERRS) including those of the mitogen-activated protein kinase (MAPK)/extracellular signal-regulated kinase (ERK) pathways, and the PI3K/Akt pathway that regulates cell proliferation, differentiation, apoptosis, invasion, and migration (Liu et al., 2015). In this study, we found that 17β-estradiol specifically decreased the levels of ERα-36 and ERα-66 in HepG2 cells. Combined with the results from a previous study (Liu et al., 2015), these data show that the reduction in ERα-36 triggers an increase in *p*-Akt levels in HepG2 cells.

Foxo3a is an important downstream signal molecule of the PI3K/Akt pathway; Foxo3a activity is strongly linked to the intracellular oxidative stress response. Activated Foxo3a reduces this response by binding to the promoters of genes encoding manganese superoxide dismutase (Mn-SOD) and catalase (Liu et al., 2018; Link, 2019). Foxo3a activity is closely related to its subcellular localization. On stimulation by growth and other factors, Akt directly phosphorylates Foxo3a, which then complexes with the 14-3-3 nuclear efflux protein to transfer from the nucleus to the cytoplasm, resulting in inhibition of transcriptional activity. Inactivation of Foxo3a transcription is common in many tumors, including those of liver, breast, and prostate cancer (Barthel et al., 2005; Fukunaga et al., 2005; Zhang et al., 2017; Link, 2019).

**FIGURE 8:**
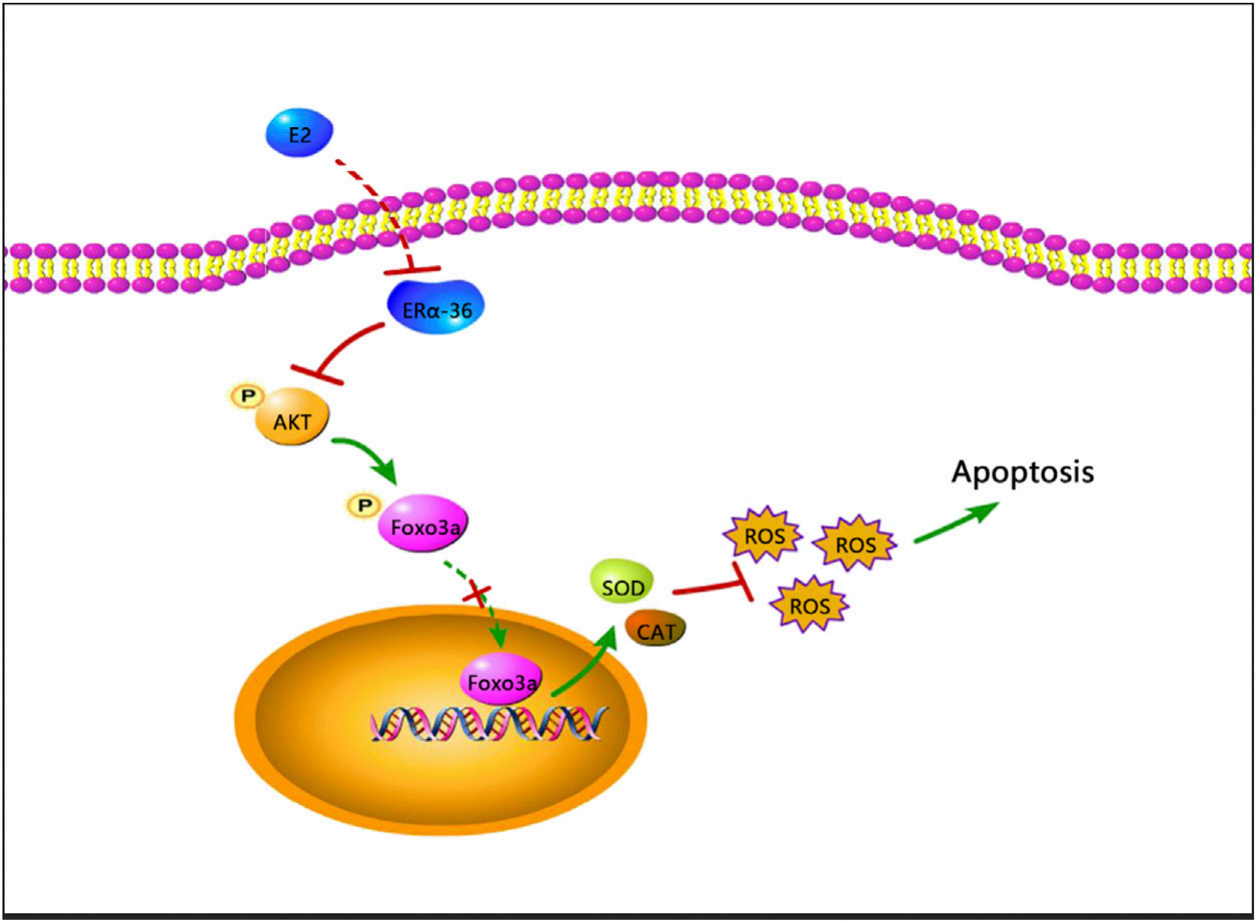
A schematic of a possible mechanism whereby apoptosis may be induced by 17β-estradiol in HepG2 cells.

We found that 17β-estradiol downregulated ERα expression in HepG2 cells and increased phosphorylation of Akt and Foxo3a, preventing nuclear entry by Foxo3a and thus the synthesis of Mn-SOD and catalase, triggering oxidative stress and ultimately apoptosis (Figure 8). To the best of our knowledge, this is the first study to investigate the role of Foxo3a phosphorylation induced by 17β-estradiol in three liver cell lines.

However, 17β-estradiol had no effect on the expression levels of ERα, phosphorylated Akt, or Foxo3a in LO2 or HepG2.2.15 cells; the mechanism of action on HepG2 cells thus remains unclear. 17β-estradiol had no effects on normal cells within a certain concentration range, but promoted apoptosis of HepG2 cells; this may be therapeutically valuable. However, 17β-estradiol therapy alone may not be ideal for liver cancer patients infected with the hepatitis B virus. The mechanism of action of 17β-estradiol should be explored further; effective treatment may also require genetic modification.

We investigated the apoptotic effects of 17β-estradiol on three different cell types and elucidated the mechanism in play. Our study provides a new theoretical basis for biotherapy of liver cancer.

## Data Availability

Illumina sequencing reads were uploaded to the SRA under accession number PRJNA664533. The rest of the data that support the conclusions of this study are available from the corresponding author upon request.
